# Quality of Life in Patients With Well-Differentiated Thyroid Carcinoma After Total Thyroidectomy in Greece

**DOI:** 10.7759/cureus.53304

**Published:** 2024-01-31

**Authors:** Olga Agglopoulou, Eleni Gkrinia, Argyro Bizaki-Vallaskangas, Jiannis Hajiioannou, Ioannis Bizakis

**Affiliations:** 1 Otolaryngology-Head and Neck Surgery, University Hospital of Larissa, Larissa, GRC; 2 Otolaryngology, Faculty of Medicine and Health Technology, University Hospital of Tampere, Tampere University, Tampere, FIN; 3 Otolaryngology-Head and Neck Surgery, Faculty of Medicine, University of Thessaly, Larissa, GRC

**Keywords:** post-thyroidectomy, complications of thyroidectomy, post-thyroidectomy complications, thyroid cancer, quality of life (qol)

## Abstract

Introduction: Well-differentiated thyroid cancer is among the most common types of endocrine cancer. Despite its increasing prevalence, the prognosis of thyroid cancer is rather good, with a five-year survival rate ranging between 80% and 90%, depending on the histological type. Not only the post-treatment complications and the side effects of the lifelong medication but also the possibility of a recurrence may have a negative impact on the patient’s quality of life (QoL).

Objective: The aim of this study is to investigate the impact of total thyroidectomy on the QoL of patients with well-differentiated thyroid carcinoma.

Methods: A prospective study was performed in patients who underwent total thyroidectomy due to well-differentiated thyroid carcinoma in the Otolaryngology-Head and Neck Surgery Department at the University Hospital of Larissa, Greece. The QoL was evaluated based on the “Quality of Life (Thyroid Version)” questionnaire.

Results: One hundred patients participated in the study, with a mean age of 51.24±15.33 years. Of these, 63 (63%) were females and 37 (37%) were males. Social concerns, physical well-being, and psychological well-being presented a gradual positive alteration during the first 12 months after the surgery, with psychological well-being reporting the most significant progress of 15.3%. However, spiritual well-being appeared to decline over time. The younger patients progressively improved their physical, psychological, and mental well-being; however, the older patients showed an amelioration of their social skills. Female patients reported higher levels of spiritual well-being, whereas males developed better social skills.

Conclusions: In the long term, total thyroidectomy can ameliorate patients’ physical status, psychological well-being, and social concerns. However, it might have a negative effect on their mental health during the first 12 months postoperatively. In addition, QoL is directly influenced by demographic characteristics such as age and gender.

## Introduction

Well-differentiated cancer of the thyroid gland is one of the most common types of endocrine neoplasias, representing the ninth most frequent malignancy worldwide [1]. During the last decades, its incidence has increased approximately by 300%, affecting a wide spectrum of patients, including adolescents and young adults, while the female-to-male ratio is estimated to be 1:2 [2,3]. Despite its increasing prevalence, the prognosis of thyroid cancer is rather good, with an estimated five-year survival rate ranging between 80% and 90% in European countries [4].

In most cases, thyroid carcinoma is diagnosed incidentally (most commonly after surgery for goiter or multinodular goiter), and symptoms such as dysphagia, neck pain, and hoarseness may appear only in advanced stages. The diagnosis is based on clinical examination, thyroid hormonal blood testing, imaging, and the results of a fine needle aspiration (FNA) biopsy [1]. Depending on the histological type and stage of the disease, management options include surgical removal of the thyroid gland, either combined with neck dissection or not, and radioactive iodine. In some cases, mild or moderate thyroid-stimulating hormone (TSH) suppressive therapy is recommended as an additional treatment [5,6]. After a total thyroidectomy, lifelong replacement of the thyroid hormone through the administration of levothyroxine is needed for all patients [5].

Both the disease and the required treatment may negatively affect patients’ physical, psychological, and mental well-being. Post-treatment complications, including side effects of the lifelong medication, as well as the risk of a recurrence, may negatively affect patients’ quality of life (QoL) [7,8]. Various questionnaires focusing on the QoL of patients with thyroid malignancy have been developed [9]. Among them, the “Quality of Life (Thyroid Version)” is a 30-item ordinal scale evaluating four separate domains: physical well-being, psychological well-being, social concerns, and spiritual well-being [10,11].

The aim of this study is to investigate the impact of total thyroidectomy on the QoL of patients with well-differentiated thyroid carcinoma.

## Materials and methods

Subjects

A prospective cohort study was conducted, including patients who underwent total thyroidectomy due to well-differentiated thyroid carcinoma at a single tertiary Otolaryngology-Head and Neck Surgery Center between January 2019 and May 2023. The study was approved by the Institutional Review Board of the University Hospital of Larissa (decision no. 9/1st/17-01-2019). All patients signed informed consent, giving permission for treatment and publishing anonymized data.

Inclusion criteria

The study included only adults (aged 18-80 years old) with well-differentiated thyroid carcinoma who underwent total thyroidectomy with or without central neck dissection. Preoperatively, the diagnosis was histologically confirmed after FNA biopsy. Patients with a history of other malignancies, previous neck surgery, histological type of thyroid carcinoma other than well-differentiated, and severe cardiac disease were omitted.

The QoL questionnaire

The assessment of QoL relied on the “Quality of Life (Thyroid Version)” questionnaire. All translations, from English to Greek and vice versa, were performed in collaboration with native Greek and English speakers. The 30-item ordinal scale is used to evaluate the QoL according to four different sectors: physical well-being (questions 1 and 2), psychological well-being (questions 3-15), social concerns (questions 16-23), and spiritual well-being (questions 24-30) [10,11]. The scoring was achieved on a scale from 0 to 10, where “0” represents “the worst outcome” and “10” represents “the best outcome.” The QoL was evaluated using the questionnaire at four different timepoints of the patient’s management: (a) preoperatively, and (b) three, (c) six, and (d) 12 months postoperatively.

Statistical analysis

The statistical analysis was performed using IBM SPSS Statistics for Windows, Version 23.0 (Released 2015; IBM Corp., Armonk, New York, United States) with both descriptive analysis and inferential analysis methods. In descriptive analysis, categorical variables were presented in counts and percentages, whereas quantitative variables were reported as a mean±standard deviation and minimum and maximum values. As a scale of reliability, we used Cronbach’s α to measure the internal consistency of different questions that constitute a parameter. A Cronbach's α value >0.7 suggests high internal consistency and reliability. Regarding the inferential analysis and the investigation of possible correlations, the Kolmogorov-Smirnov test was used to check the normality of parameter distribution for the sample of this study. Furthermore, Spearman's rank correlation (rho) and Mann-Whitney U test were also performed. The significance level was set at *5% and **1%.

## Results

Demographics

Based on the inclusion and exclusion criteria, 118 patients were considered eligible; 100 completed all follow-up questionnaires and represented the study cohort. Among them, 63 were females. The mean age was estimated at 51.24±15.33 years (range 18-80 years). Ninety-six patients were diagnosed with well-differentiated papillary carcinoma and four with well-differentiated follicular carcinoma. In addition to the total thyroidectomy that was performed on all the patients, 32 of 100 patients also underwent neck dissection.

Quality of life

General Assessment of QoL

**Figure 1:**
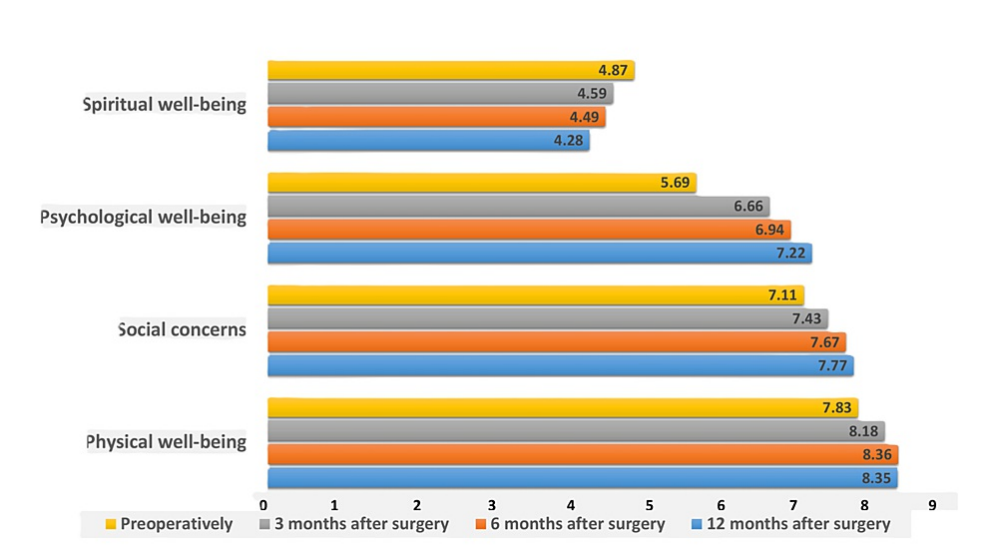
Alteration of parameters of QoL. QoL: quality of life.

Physical well-being presented significant improvement at three months and continued to rise up to six months postoperatively. Psychological well-being appeared to upgrade gradually up to 12 months, and during the same period, social concerns showed enhancement. Spiritual well-being decreased over time (Figure 1). Generally, the QoL improved significantly at 12 months compared to the preoperative period (QoL index changed from 6.53 to 7.25, p<0.05).

Preoperatively, all parameters, except for spiritual well-being, indicated high internal consistency. The higher level of QoL was presented in physical well-being (7.83±1.26) and social concerns (7.11±1.37), whereas the lowest level was recorded in psychological well-being (5.69±1.17) and spiritual well-being (4.87±1.24) (Table 1). A positive correlation was detected among physical well-being, psychological well-being, social concerns, and QoL, while physical well-being was negatively associated with spiritual well-being (Table 2). Age was positively correlated with social concerns, and male gender was positively correlated with physical well-being, social concerns, and QoL (Tables 3, 4).

**Table 1:** Analysis of QoL (Thyroid Version). QoL: quality of life.

	Preoperatively		
QoL-Thyroid	Cronbach's α	Mean±SD	Minimum-maximum
Physical well-being	0.859	7.83±1.26	2.69-10
Psychological well-being	0.854	5.69±1.17	2.19–8.88
Social concerns	0.887	7.11±1.37	2.14–9.29
Spiritual well-being	0.665	4.87±1.24	2.86–8.57
Total QoL	0.894	6.53±0.90	3.14–8.34
	Three months after surgery		
QoL-Thyroid	Cronbach's α	Mean±SD	Minimum-maximum
Physical well-being	0.827	8.18±0.77	4.23–9.62
Psychological well-being	0.713	6.66±0.67	3.59–7.95
Social concerns	0.846	7.43±0.79	3.86–8.71
Spiritual well-being	0.687	4.59±0.85	2.86–7.14
Total QoL	0.806	6.95±0.47	4.80–7.84
	Six months after surgery		
QoL-Thyroid	Cronbach's α	Mean±SD	Minimum-maximum
Physical well-being	0.859	8.36±0.66	6.15–9.92
Psychological well-being	0.784	6.94±0.57	4.82–8.36
Social concerns	0.705	7.67±0.47	6.64–8.57
Spiritual well-being	0.754	4.49±0.87	3.14–6.71
Total QoL	0.838	7.14±0.39	5.71–7.79
	12 months after surgery		
QOL-Thyroid	Cronbach's α	Mean±SD	Minimum-maximum
Physical well-being	0.775	8.35±0.49	6.85–9.85
Psychological well-being	0.714	7.22±0.44	4.82–8.36
Social concerns	0.616	777±0.39	6.93–8.93
Spiritual well-being	0.665	4.28±0.70	2.86–6.71
Total QoL	0.717	7.25±0.29	6.43–8.11

**Table 2:** Associations of QoL factors before and after total thyroidectomy. QoL: quality of life. *5% Level of significance. **1% Level of significance.

		Preoperatively			
		Physical well-being	Psychological well-being	Social concerns	Spiritual well-being
Psychological well-being	rho	0.489^**^			
	p	<0.001			
Social concerns	rho	0.470^**^	0.473^**^		
	p	<0.001	<0.001		
Spiritual well-being	rho	-0.201^*^	0.032	-0.136	
	p	0.045	0.75	0.179	
Total QoL	rho	0.716^**^	0.822^**^	0.745^**^	0.064
	p	<0.001	<0.001	<0.001	0.53
		Three months after surgery			
		Physical well-being	Psychological well-being	Social concerns	Spiritual well-being
Psychological well-being	rho	0.277^**^			
	p	0.005			
Social concerns	rho	-0.02	0.263^**^		
	p	0.842	0.008		
Spiritual well-being	rho	-0.142	-0.041	-0.238^*^	
	p	0.159	0.685	0.017	
Total QoL	rho	0.488^**^	0.820^**^	0.546^**^	0.046
	p	<0.001	<0.001	<0.001	0.65
		Six months after surgery			
		Physical well-being	Psychological well-being	Social concerns	Spiritual well-being
Psychological well-being	rho	0.481^**^			
	p	<0.001			
Social concerns	rho	0.163	0.334^**^		
	p	0.105	0.001		
Spiritual well-being	rho	0.168	0.112	-0.051	
	p	0.095	0.267	0.618	
Total QoL	rho	0.701^**^	0.854^**^	0.504^**^	0.352^**^
	p	<0.001	<0.001	<0.001	<0.001
		12 months after surgery			
		Physical well-being	Psychological well-being	Social concerns	Spiritual well-being
Psychological well-being	rho	0.552^**^			
	p	<0.001			
Social concerns	rho	0.096	0.200^*^		
	p	0.342	0.046		
Spiritual well-being	rho	0.083	0.047	-0.128	
	p	0.41	0.642	0.205	
Total QoL	rho	0.741^**^	0.823^**^	0.394^**^	0.321^**^
	p	<0.001	<0.001	<0.001	0.001

**Table 3:** Effect of patient's age on QoL factors before and after total thyroidectomy. QoL: quality of life. *5% Level of significance. **1% Level of significance.

	Preoperatively	
		Age
Physical well-being	rho	0.09
	p	0.372
Psychological well-being	rho	-0.005
	p	0.962
Social concerns	rho	0.404^**^
	p	<0.001
Spiritual well-being	rho	-0.173
	p	0.086
Total QoL	rho	0.105
	p	0.298
	Three months after surgery	
		Age
Physical Well-being	rho	-0.325^**^
	p	0.001
Psychological well-being	rho	-0.041
	p	0.687
Social concerns	rho	0.537^**^
	p	<0.001
Spiritual well-being	rho	-0.109
	p	0.281
Total QoL	rho	0.095
	p	0.35
	Six months after surgery	
		Age
Physical Well-being	rho	-0.462^**^
	p	<0.001
Psychological well-being	rho	-0.188
	p	0.06
Social concerns	rho	0.324^**^
	p	0001
Spiritual well-being	rho	-0.251^*^
	p	0.012
Total QoL	rho	-0.213^*^
	p	0.034
	12 months after surgery	
		Age
Physical well-being	rho	-0.408^**^
	p	<0.001
Psychological well-being	rho	-0.238^*^
	p	0.017
Social concerns	rho	0.484^**^
	p	<0.001
Spiritual well-being	rho	-0.210^*^
	p	0.036
Total QoL	rho	-0.213^*^
	p	0.033

**Table 4:** Effect of patient's gender on QoL factors before and after total thyroidectomy. QoL: quality of life.

	Preoperatively		
	Gender		p value
	Male	Female	
Physical well-being	8.38	7.92	0.007
Psychological well-being	6.06	5.43	0.002
Social concerns	7.64	7.42	0.037
Spiritual well-being	4.28	4.57	0.333
Total QoL	6.84	6.52	0.011
	Three months after surgery		
	Gender		p value
	Male	Female	
Physical well-being	8.46	8.23	0.168
Psychological well-being	6.72	6.72	0.98
Social concerns	7.64	7.57	0.479
Spiritual well-being	4.28	4.57	0.095
Total QoL	7.01	7	0.563
	Six months after surgery		
	Gender		p value
	Male	Female	
Physical well-being	8.53	8.53	0.277
Psychological well-being	7	7.04	0.516
Social concerns	7.85	7.71	0.197
Spiritual well-being	4.14	4.42	0.012
Total QoL	7.26	7.21	0.477
	12 months after surgery		
	Gender		p value
	Male	Female	
Physical well-being	8.46	8.3	0.075
Psychological well-being	7.22	7.13	0.175
Social concerns	7.85	7.57	0.004
Spiritual well-being	3.85	4.42	<0.001
Total QoL	7.26	7.19	0.206

At three months after total thyroidectomy, physical well-being (8.18±0.77) and social concerns (7.43±0.79) presented the highest levels, while psychological well-being (6.66±0.67) and spiritual well-being (4.59±0.85) presented the lowest (Table 1). A positive correlation was detected among physical well-being, psychological well-being, and general QoL, whereas social concerns were negatively correlated with spiritual well-being (Table 2). The age was negatively connected to social concerns and physical well-being (Tables 3, 4).

At six months, all four parameters were characterized by high reliability, with physical well-being (8.36±0.66) and social concerns (7.67±0.47) presenting the highest levels (Table 1). Positive correlations were detected among physical well-being, social concerns, psychological well-being, and general QoL (Table 2). The age was positively associated with social concerns and negatively with physical well-being, spiritual well-being, and QoL (Table 3). Females presented higher spiritual well-being compared to males (Table 4).

Only physical (8.35±0.49) and psychological well-being (7.22±0.44) resulted in high reliability at 12 months postoperatively (Table 1). A positive correlation was detected among physical well-being, psychological well-being, social concerns, and QoL (Table 2). The age was positively related to social concerns but negatively correlated with physical well-being, psychological well-being, spiritual well-being, and QoL (Table 3). Males were positively connected to social concerns, whereas females were positively connected to spiritual well-being (Table 4).

## Discussion

According to this analysis, all parameters, apart from spiritual well-being, presented a gradual increase during the initial 12 months after surgery, with psychological well-being showing significant progress with a rise estimated at 15.3%. On the other hand, spiritual well-being appeared to decrease throughout the follow-up. This could be justified by the fact that the study was conducted during the COVID-19 pandemic. Therefore, the patients, except those with cancer, had to confront additional psychological stress. Dehghan et al., in their study, reported that patients diagnosed with thyroid cancer during the COVID-19 pandemic developed high levels of physical and mental stress that were also connected to their preoccupation about SARS-CoV-2 contamination [12]. Furthermore, Khiyali et al. confirmed a significant inverse correlation between the spiritual well-being of oncological patients and COVID-19-related anxiety [13].

A strong direct connection among physical well-being, psychological well-being, social concerns, and total QoL levels was observed in this analysis, not only preoperatively but also during the 12-month follow-up period. On the contrary, spiritual well-being was inversely correlated with physical well-being before the surgery and also with social concerns three months after total thyroidectomy. Younger patients appeared progressive improvement in terms of physical, psychological, and mental well-being, whereas older patients showed incremental amelioration of their social concerns and skills. Females reported higher levels of spiritual well-being, while males developed better social skills. Previous studies’ findings are in accordance with the current analysis, indicating that younger patients and males show lower anxiety and depression levels and, subsequently, better QoL [14-16]. Other socioeconomic factors, such as weak financial state and unemployment, have been strongly associated with higher distress and lower QoL [17,18], while the role of superior education and marital status on QoL seems rather controversial [14,19-21].

However, the QoL may be influenced by various factors, related to the nature of the disease or not. Disease-specific characteristics, such as cancer stage, histologic type, disease status at the time of diagnosis, or pre-existing comorbidities, may affect the QoL among thyroid cancer survivors [14,22-24]. Furthermore, patient’s mental status and QoL may be impaired by the management approach and therapy’s side effects [25-27]. Good communication between thyroid cancer survivors and healthcare providers, in addition to psychological and behavioral support, is mandatory to improve patient’s negative perceptions regarding potential complications, metastasis, or recurrence [28,29].

Limitations

The present study is characterized by several limitations, hampering robust conclusions. The “Quality of Life-Thyroid Version” questionnaire was officially translated from English to Greek (forward and backward translation), and preoperatively, all parameters, apart from spiritual well-being, indicated high internal consistency. However, no validation has been performed among the Greek population. The small sample size affects the risk of bias, and it does not permit widely applicable conclusions. Factors such as pre-existing comorbidities, stage of disease, and type of management were not evaluated, adding further bias. Further long-scale studies are necessary to detect further correlations among the different parameters that constitute QoL and various socioeconomic and factors, in order to improve the management protocols and procedures of these patients.

## Conclusions

Well-differentiated thyroid cancer may affect patients’ QoL. Total thyroidectomy seems to improve patients’ physical status, psychological well-being, and social concerns. A negative effect on mental health should be acknowledged within the 12-month follow-up. 
